# Correction: A robust iron catalyst for the selective hydrogenation of substituted (iso)quinolones

**DOI:** 10.1039/c8sc90188k

**Published:** 2018-09-28

**Authors:** Basudev Sahoo, Carsten Kreyenschulte, Giovanni Agostini, Henrik Lund, Stephan Bachmann, Michelangelo Scalone, Kathrin Junge, Matthias Beller

**Affiliations:** a Leibniz-Institut für Katalyse e.V. an der Universität Rostock , Albert-Einstein-Str. 29a , 18059 Rostock , Germany . Email: matthias.beller@catalysis.de; b Process Chemistry and Catalysis, F. Hoffmann-La Roche Ltd. , Grenzacherstrasse 124 , 4070 Basel , Switzerland

## Abstract

Correction for ‘A robust iron catalyst for the selective hydrogenation of substituted (iso)quinolones’ by Basudev Sahoo *et al.*, *Chem. Sci.*, 2018, DOI: ; 10.1039/c8sc02744g.



## 

The authors regret that the term “(iso)quinolones” was used throughout the article, including the title, when the correct term should be “(iso)quinolines”.

The Royal Society of Chemistry apologises for these errors and any consequent inconvenience to authors and readers.

